# 16-Isopropyl-5,9-dimethyl­tetra­cyclo­[10.2.2.0^1,10^.0^4,9^]hexa­dec-15-ene-5,13,14-tricarboxylic acid dimethyl­formamide disolvate

**DOI:** 10.1107/S1600536810016594

**Published:** 2010-05-12

**Authors:** Xu Xu, Zhan-qian Song, Hong-xiao Wang, Shi-bin Shang

**Affiliations:** a Institute of Chemical Industry of Forest Products, Chinese Academy of Forestry, Nanjing 210042, People’s Republic of China

## Abstract

The title compound, C_24_H_34_O_6_·2C_3_H_7_NO, which was isolated from fumaric-modified rosin, has four asymmetrically fused six-membered rings and three carboxylic acid substituents. It contains two fused and unbridged cyclo­hexane rings, which form a *trans* ring junction with a chair conformation. The asymmetric unit includes one fumaropimaric acid and two dimethyl­formamide mol­ecules. The crystal structure is stabilized through inter­molecular O—H⋯O hydrogen bonds between dimethyl­formamide and fumaropimaric acid.

## Related literature

For various applications of rosin, see: Halbrook & Lawrence (1958 ▶). For the separation of the title compound, see: Aldrich (1971 ▶); Halbrook & Lawrence (1959 ▶); Song *et al.* (2009 ▶).
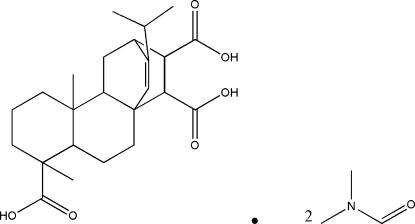


## Experimental

### 

#### Crystal data

C_24_H_34_O_6_·2C_3_H_7_NO
*M* *_r_* = 564.70
Orthorhombic, 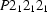

*a* = 7.1260 (14) Å
*b* = 11.342 (2) Å
*c* = 39.610 (8) Å
*V* = 3201.4 (11) Å^3^
*Z* = 4
Mo *K*α radiation
μ = 0.08 mm^−1^
*T* = 296 K
0.30 × 0.20 × 0.10 mm

#### Data collection

Enraf–Nonius CAD-4 diffractometer
Absorption correction: ψ scan (North *et al.*, 1968 ▶) *T*_min_ = 0.975, *T*_max_ = 0.992
5821 measured reflections
3355 independent reflections
2039 reflections with *I* > 2σ(*I*)
*R*_int_ = 0.056
3 standard reflections every 200 reflections  intensity decay: 1%

#### Refinement

*R*[*F* ^2^ > 2σ(*F* ^2^)] = 0.066
*wR*(*F* ^2^) = 0.182
*S* = 0.99
3355 reflections
355 parameters
1 restraint
H-atom parameters constrained
Δρ_max_ = 0.38 e Å^−3^
Δρ_min_ = −0.37 e Å^−3^

### 

Data collection: *CAD-4 EXPRESS* (Enraf–Nonius, 1994 ▶); cell refinement: *CAD-4 EXPRESS*; data reduction: *XCAD4* (Harms & Wocadlo, 1995 ▶); program(s) used to solve structure: *SHELXS97* (Sheldrick, 2008 ▶); program(s) used to refine structure: *SHELXL97* (Sheldrick, 2008 ▶); molecular graphics: *ORTEP-3* (Farrugia, 1997 ▶); software used to prepare material for publication: *SHELXTL* (Sheldrick, 2008 ▶).

## Supplementary Material

Crystal structure: contains datablocks I, global. DOI: 10.1107/S1600536810016594/bq2203sup1.cif

Structure factors: contains datablocks I. DOI: 10.1107/S1600536810016594/bq2203Isup2.hkl

Additional supplementary materials:  crystallographic information; 3D view; checkCIF report

## Figures and Tables

**Table 1:** Hydrogen-bond geometry (Å, °)

*D*—H⋯*A*	*D*—H	H⋯*A*	*D*⋯*A*	*D*—H⋯*A*
O2—H2*A*⋯O7^i^	0.82	1.85	2.653 (8)	168
O4—H4*B*⋯O8^ii^	0.82	1.74	2.549 (6)	168
O5—H5*A*⋯O3^iii^	0.82	1.96	2.781 (5)	176

Symmetry codes: (i) 
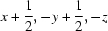
; (ii) 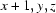; (iii) 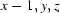.

**Fig. 1.**
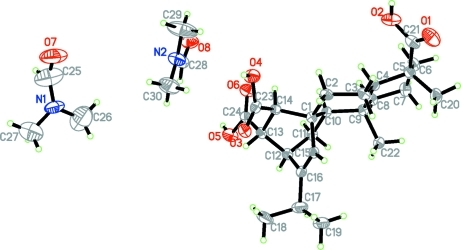
A view of the molecular structure of (I), showing displacement ellipsoids at the 25% probability level.

**Fig. 2.**
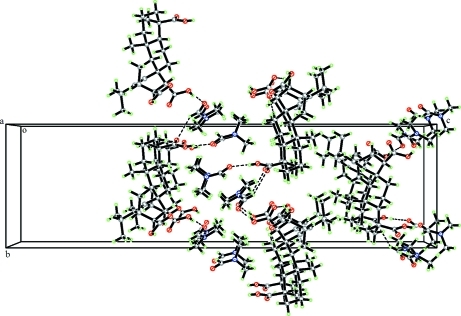
A view of the packing of the title compound.

## supplementary crystallographic information

### Comment

As an abundant and renewable material, rosin is mainly known as additives and modifiers for various applications (Halbrook *et al.*, 1958). Fumaric modified rosin is one of modified products of rosin. The title compound has been isolated by solvent extracting (Aldrich *et al.*, 1971) and solvent washing (Halbrook *et al.*, 1959) from fumaric modified rosin, but there are some problems such as complicated operation and large amounts of toxic organic solvents. Therefore, a new method has been used to separate the title compound (Song *et al.*,  2009). In this work, we describe the crystal structure of the title compound (I). The molecular structure is shown in Fig. 1 and the crystal packing in Fig. 2.

### Experimental

The fumaric modified rosin (10 g) was dissolved in ethyl alcohol, then 5% sodium hydroxide solution (30 mL) and 2% aqueous sodium chloride solution (500 ml) was added dropwise successively with constant stirring. After dropping the mixture was stirred for another 15 minutes and then filtered. The filtrate was adjusted pH to 3 using 5% hydrochloric acid solution. The title compound was precipitated from the solution. Crystals of the title compound suitable for X-ray diffraction were obtained by slow evaporation of DMF solution. The crystal data were collected on an Enraf–Nonius CAD-4 diffractometer. Data collection and cell refinement were performed using Enraf–Nonius *CAD-4 Software*.

### Refinement

All H atoms bonded to the C atoms and O atoms were placed geometrically at the distances of 0.93-0.98 Å and 0.82 Å respectively, and included in the refinement in riding motion approximation with U_iso_(H) = 1.2 or 1.5U_eq_ of the carrier atom. 2466 Friedel pairs were averaged before the final refinement as the absolute configuration could not be determined unambiguously.

### Crystal data

**Table:**

C_24_H_34_O_6_·2C_3_H_7_NO	*F*(000) = 1224
*M**_r_* = 564.70	*D*_x_ = 1.172 Mg m^−^^3^
Orthorhombic, *P*2_1_2_1_2_1_	Mo *K*α radiation, λ = 0.71073 Å
Hall symbol: P2ac2ab	Cell parameters from 25 reflections
*a* = 7.1260 (14) Å	θ = 9–12°
*b* = 11.342 (2) Å	µ = 0.08 mm^−^^1^
*c* = 39.610 (8) Å	*T* = 296 K
*V* = 3201.4 (11) Å^3^	Block, colorless
*Z* = 4	0.30 × 0.20 × 0.10 mm

### Data collection

**Table:**

Enraf–Nonius CAD-4 diffractometer	2039 reflections with *I* > 2σ(*I*)
Radiation source: fine-focus sealed tube	*R*_int_ = 0.056
graphite	θ_max_ = 25.3°, θ_min_ = 1.0°
ω/2θ scans	*h* = −8→8
Absorption correction: ψ scan (North et al., 1968)	*k* = 0→13
*T*_min_ = 0.975, *T*_max_ = 0.992	*l* = 0→47
5821 measured reflections	3 standard reflections every 200 reflections
3355 independent reflections	intensity decay: 1%

### Refinement

**Table:**

Refinement on *F*^2^	Primary atom site location: structure-invariant direct methods
Least-squares matrix: full	Secondary atom site location: difference Fourier map
*R*[*F*^2^ > 2σ(*F*^2^)] = 0.066	Hydrogen site location: inferred from neighbouring sites
*wR*(*F*^2^) = 0.182	H-atom parameters constrained
*S* = 0.99	*w* = 1/[σ^2^(*F*_o_^2^) + (0.1012*P*)^2^] where *P* = (*F*_o_^2^ + 2*F*_c_^2^)/3
3355 reflections	(Δ/σ)_max_ < 0.001
355 parameters	Δρ_max_ = 0.38 e Å^−^^3^
1 restraint	Δρ_min_ = −0.37 e Å^−^^3^

### Special details

**Table:**

Geometry. All esds (except the esd in the dihedral angle between two l.s. planes) are estimated using the full covariance matrix. The cell esds are taken into account individually in the estimation of esds in distances, angles and torsion angles; correlations between esds in cell parameters are only used when they are defined by crystal symmetry. An approximate (isotropic) treatment of cell esds is used for estimating esds involving l.s. planes.
Refinement. Refinement of *F*^2^ against ALL reflections. The weighted *R*-factor wR and goodness of fit *S* are based on *F*^2^, conventional *R*-factors *R* are based on *F*, with *F* set to zero for negative *F*^2^. The threshold expression of *F*^2^ > σ(*F*^2^) is used only for calculating *R*-factors(gt) etc. and is not relevant to the choice of reflections for refinement. *R*-factors based on *F*^2^ are statistically about twice as large as those based on *F*, and *R*- factors based on ALL data will be even larger.

### Fractional atomic coordinates and isotropic or equivalent isotropic displacement parameters (Å^2^)

**Table:**

	*x*	*y*	*z*	*U*_iso_*/*U*_eq_	
O1	1.0202 (7)	−0.3704 (5)	0.10397 (15)	0.0998 (17)	
O2	0.7495 (8)	−0.3154 (4)	0.08207 (11)	0.0955 (16)	
H2A	0.8053	−0.3257	0.0642	0.143*	
O3	0.8126 (5)	0.3478 (3)	0.11878 (9)	0.0512 (9)	
O4	0.7388 (7)	0.2389 (3)	0.07384 (9)	0.0740 (12)	
H4B	0.8281	0.2735	0.0653	0.111*	
O5	0.1597 (5)	0.3461 (4)	0.15090 (11)	0.0789 (13)	
H5A	0.0552	0.3488	0.1423	0.118*	
O6	0.2060 (6)	0.2170 (4)	0.10993 (11)	0.0724 (12)	
C1	0.6924 (6)	0.0877 (4)	0.14378 (11)	0.0376 (10)	
C2	0.8335 (7)	0.0249 (4)	0.12060 (12)	0.0446 (12)	
H2B	0.7764	0.0130	0.0986	0.053*	
H2C	0.9425	0.0750	0.1176	0.053*	
C3	0.8951 (7)	−0.0909 (4)	0.13419 (13)	0.0462 (12)	
H3A	0.9605	−0.0792	0.1554	0.055*	
H3B	0.9814	−0.1277	0.1185	0.055*	
C4	0.7256 (7)	−0.1714 (4)	0.13976 (12)	0.0416 (11)	
H4A	0.6524	−0.1637	0.1189	0.050*	
C5	0.7738 (7)	−0.3056 (4)	0.14164 (13)	0.0497 (13)	
C6	0.5870 (8)	−0.3741 (5)	0.14560 (15)	0.0602 (15)	
H6A	0.5148	−0.3663	0.1249	0.072*	
H6B	0.6147	−0.4571	0.1488	0.072*	
C7	0.4702 (8)	−0.3317 (4)	0.17471 (16)	0.0579 (15)	
H7A	0.5385	−0.3438	0.1956	0.069*	
H7B	0.3552	−0.3773	0.1758	0.069*	
C8	0.4223 (7)	−0.2011 (4)	0.17099 (14)	0.0482 (13)	
H8A	0.3506	−0.1762	0.1905	0.058*	
H8B	0.3434	−0.1910	0.1512	0.058*	
C9	0.5950 (7)	−0.1214 (4)	0.16751 (12)	0.0405 (11)	
C10	0.5277 (6)	0.0027 (4)	0.15488 (11)	0.0372 (11)	
H10A	0.4542	−0.0122	0.1344	0.045*	
C11	0.3962 (7)	0.0695 (4)	0.17891 (13)	0.0455 (12)	
H11A	0.3951	0.0309	0.2008	0.055*	
H11B	0.2694	0.0683	0.1700	0.055*	
C12	0.4619 (7)	0.1977 (4)	0.18299 (13)	0.0445 (12)	
H12A	0.3805	0.2399	0.1989	0.053*	
C13	0.4592 (7)	0.2585 (4)	0.14818 (12)	0.0456 (12)	
H13A	0.5121	0.3377	0.1509	0.055*	
C14	0.5889 (7)	0.1887 (4)	0.12420 (12)	0.0401 (11)	
H14A	0.5091	0.1514	0.1071	0.048*	
C15	0.7754 (7)	0.1409 (4)	0.17497 (12)	0.0396 (11)	
H15A	0.9027	0.1341	0.1797	0.048*	
C16	0.6604 (7)	0.1978 (4)	0.19544 (12)	0.0463 (12)	
C17	0.7100 (11)	0.2617 (6)	0.22769 (16)	0.0840 (15)	
H17A	0.6214	0.2370	0.2452	0.101*	
C18	0.6869 (14)	0.3950 (5)	0.22221 (16)	0.094 (2)	
H18A	0.5622	0.4111	0.2143	0.140*	
H18B	0.7764	0.4215	0.2058	0.140*	
H18C	0.7075	0.4356	0.2431	0.140*	
C19	0.9084 (10)	0.2325 (6)	0.23979 (15)	0.0840 (15)	
H19A	0.9205	0.1488	0.2427	0.126*	
H19B	0.9315	0.2712	0.2609	0.126*	
H19C	0.9979	0.2593	0.2234	0.126*	
C20	0.9122 (8)	−0.3388 (5)	0.16926 (16)	0.0671 (16)	
H20A	1.0260	−0.2946	0.1664	0.101*	
H20B	0.9396	−0.4216	0.1679	0.101*	
H20C	0.8586	−0.3214	0.1909	0.101*	
C21	0.8668 (10)	−0.3355 (5)	0.10798 (18)	0.0660 (17)	
C22	0.6957 (8)	−0.1143 (4)	0.20269 (12)	0.0499 (13)	
H22A	0.7342	−0.1918	0.2095	0.075*	
H22B	0.6107	−0.0824	0.2191	0.075*	
H22C	0.8039	−0.0642	0.2009	0.075*	
C23	0.7250 (7)	0.2684 (5)	0.10591 (12)	0.0437 (12)	
C24	0.2656 (7)	0.2712 (4)	0.13405 (13)	0.0468 (12)	
N1	0.3130 (14)	0.9174 (8)	0.03150 (17)	0.120 (3)	
O7	0.3811 (15)	0.8410 (9)	−0.01985 (18)	0.187 (4)	
C25	0.310 (2)	0.8890 (15)	0.0001 (3)	0.195 (6)	
H25A	0.2045	0.9239	−0.0094	0.234*	
C26	0.244 (2)	0.8464 (12)	0.0571 (4)	0.206 (6)	
H26A	0.2250	0.7680	0.0486	0.309*	
H26B	0.1266	0.8776	0.0650	0.309*	
H26C	0.3322	0.8444	0.0754	0.309*	
C27	0.343 (3)	1.0316 (11)	0.0438 (3)	0.216 (7)	
H27A	0.3759	1.0827	0.0254	0.325*	
H27B	0.4431	1.0304	0.0600	0.325*	
H27C	0.2305	1.0598	0.0543	0.325*	
O8	0.0025 (8)	0.3316 (5)	0.03938 (11)	0.0894 (15)	
N2	0.2801 (9)	0.4273 (5)	0.04118 (14)	0.0842 (17)	
C28	0.1104 (10)	0.4000 (5)	0.05282 (15)	0.0647 (16)	
H28A	0.0714	0.4360	0.0727	0.078*	
C29	0.3562 (15)	0.3693 (11)	0.0120 (3)	0.181 (6)	
H29A	0.2611	0.3211	0.0018	0.271*	
H29B	0.4600	0.3206	0.0187	0.271*	
H29C	0.3983	0.4273	−0.0039	0.271*	
C30	0.3990 (13)	0.5018 (7)	0.0597 (2)	0.123 (3)	
H30A	0.3318	0.5335	0.0787	0.185*	
H30B	0.4412	0.5651	0.0456	0.185*	
H30C	0.5052	0.4577	0.0676	0.185*	

### Atomic displacement parameters (Å^2^)

**Table:**

	*U*^11^	*U*^22^	*U*^33^	*U*^12^	*U*^13^	*U*^23^
O1	0.075 (3)	0.099 (4)	0.126 (4)	0.022 (3)	0.030 (3)	−0.025 (3)
O2	0.109 (4)	0.117 (4)	0.061 (3)	−0.005 (4)	0.008 (3)	−0.027 (3)
O3	0.0382 (18)	0.0476 (19)	0.068 (2)	−0.0019 (18)	−0.0004 (18)	−0.0041 (18)
O4	0.092 (3)	0.075 (3)	0.054 (2)	−0.025 (3)	0.014 (2)	0.001 (2)
O5	0.045 (2)	0.092 (3)	0.099 (3)	0.023 (2)	−0.019 (2)	−0.032 (3)
O6	0.048 (2)	0.083 (3)	0.086 (3)	0.005 (2)	−0.014 (2)	−0.025 (3)
C1	0.031 (2)	0.041 (2)	0.042 (2)	−0.003 (2)	0.006 (2)	0.001 (2)
C2	0.044 (3)	0.045 (3)	0.045 (3)	−0.002 (2)	0.012 (2)	−0.007 (2)
C3	0.038 (2)	0.047 (3)	0.054 (3)	−0.005 (2)	0.006 (2)	−0.013 (2)
C4	0.039 (2)	0.041 (2)	0.045 (3)	0.004 (2)	−0.001 (2)	−0.004 (2)
C5	0.046 (3)	0.047 (3)	0.057 (3)	0.005 (3)	−0.002 (3)	−0.007 (2)
C6	0.059 (3)	0.042 (3)	0.080 (4)	0.002 (3)	−0.003 (3)	−0.011 (3)
C7	0.049 (3)	0.043 (3)	0.081 (4)	−0.013 (3)	0.004 (3)	0.010 (3)
C8	0.039 (3)	0.041 (3)	0.065 (3)	−0.001 (2)	−0.002 (3)	−0.004 (2)
C9	0.032 (2)	0.042 (2)	0.047 (3)	−0.001 (2)	0.003 (2)	−0.001 (2)
C10	0.035 (2)	0.036 (2)	0.041 (3)	−0.002 (2)	−0.001 (2)	0.002 (2)
C11	0.034 (2)	0.048 (3)	0.054 (3)	0.004 (2)	0.006 (2)	0.003 (2)
C12	0.040 (3)	0.047 (3)	0.046 (3)	0.008 (2)	0.009 (2)	0.002 (2)
C13	0.040 (3)	0.037 (2)	0.060 (3)	0.001 (2)	−0.003 (3)	−0.004 (2)
C14	0.037 (2)	0.041 (2)	0.042 (3)	−0.003 (2)	−0.003 (2)	−0.002 (2)
C15	0.033 (2)	0.038 (2)	0.048 (3)	0.005 (2)	−0.006 (2)	0.001 (2)
C16	0.051 (3)	0.042 (3)	0.046 (3)	−0.001 (3)	−0.009 (2)	0.003 (2)
C17	0.093 (3)	0.094 (3)	0.065 (3)	0.007 (3)	−0.025 (3)	−0.023 (3)
C18	0.145 (7)	0.065 (4)	0.071 (4)	−0.015 (5)	0.018 (5)	−0.027 (3)
C19	0.093 (3)	0.094 (3)	0.065 (3)	0.007 (3)	−0.025 (3)	−0.023 (3)
C20	0.056 (3)	0.059 (4)	0.085 (4)	0.014 (3)	−0.014 (3)	−0.001 (3)
C21	0.073 (4)	0.050 (3)	0.074 (4)	−0.002 (3)	0.009 (4)	−0.015 (3)
C22	0.049 (3)	0.051 (3)	0.050 (3)	0.004 (3)	−0.008 (3)	−0.001 (2)
C23	0.039 (3)	0.051 (3)	0.042 (3)	0.008 (3)	0.004 (2)	0.008 (2)
C24	0.041 (3)	0.043 (3)	0.056 (3)	−0.002 (3)	−0.003 (3)	0.002 (2)
N1	0.162 (7)	0.141 (6)	0.057 (4)	−0.015 (6)	−0.022 (5)	−0.017 (4)
O7	0.226 (10)	0.244 (9)	0.091 (5)	0.046 (9)	−0.020 (6)	−0.070 (5)
C25	0.176 (13)	0.296 (19)	0.114 (9)	0.005 (14)	−0.001 (10)	−0.042 (12)
C26	0.197 (14)	0.228 (15)	0.192 (13)	−0.035 (14)	−0.023 (13)	0.053 (11)
C27	0.31 (2)	0.160 (11)	0.183 (12)	−0.016 (15)	−0.052 (14)	−0.039 (9)
O8	0.101 (4)	0.105 (4)	0.061 (3)	−0.035 (3)	0.016 (3)	−0.008 (2)
N2	0.085 (4)	0.087 (4)	0.081 (4)	−0.016 (4)	0.010 (4)	−0.025 (3)
C28	0.075 (4)	0.063 (4)	0.056 (4)	−0.002 (4)	0.004 (3)	−0.004 (3)
C29	0.144 (10)	0.231 (12)	0.168 (10)	−0.063 (10)	0.086 (8)	−0.114 (9)
C30	0.104 (6)	0.121 (6)	0.143 (7)	−0.025 (6)	−0.018 (6)	−0.043 (6)

### Geometric parameters (Å, °)

**Table:**

O1—C21	1.174 (7)	C13—C14	1.543 (7)
O2—C21	1.343 (8)	C13—H13A	0.9800
O2—H2A	0.8200	C14—C23	1.511 (7)
O3—C23	1.209 (6)	C14—H14A	0.9800
O4—C23	1.317 (6)	C15—C16	1.321 (7)
O4—H4B	0.8200	C15—H15A	0.9300
O5—C24	1.317 (6)	C16—C17	1.511 (7)
O5—H5A	0.8200	C17—C19	1.529 (9)
O6—C24	1.213 (6)	C17—C18	1.536 (9)
C1—C15	1.496 (6)	C17—H17A	0.9800
C1—C2	1.537 (6)	C18—H18A	0.9600
C1—C14	1.568 (6)	C18—H18B	0.9600
C1—C10	1.581 (6)	C18—H18C	0.9600
C2—C3	1.486 (7)	C19—H19A	0.9600
C2—H2B	0.9700	C19—H19B	0.9600
C2—H2C	0.9700	C19—H19C	0.9600
C3—C4	1.531 (6)	C20—H20A	0.9600
C3—H3A	0.9700	C20—H20B	0.9600
C3—H3B	0.9700	C20—H20C	0.9600
C4—C9	1.547 (6)	C22—H22A	0.9600
C4—C5	1.562 (6)	C22—H22B	0.9600
C4—H4A	0.9800	C22—H22C	0.9600
C5—C20	1.520 (7)	N1—C25	1.284 (13)
C5—C21	1.527 (8)	N1—C26	1.385 (13)
C5—C6	1.549 (7)	N1—C27	1.399 (12)
C6—C7	1.501 (8)	O7—C25	1.085 (14)
C6—H6A	0.9700	C25—H25A	0.9300
C6—H6B	0.9700	C26—H26A	0.9600
C7—C8	1.527 (7)	C26—H26B	0.9600
C7—H7A	0.9700	C26—H26C	0.9600
C7—H7B	0.9700	C27—H27A	0.9600
C8—C9	1.533 (6)	C27—H27B	0.9600
C8—H8A	0.9700	C27—H27C	0.9600
C8—H8B	0.9700	O8—C28	1.215 (7)
C9—C22	1.569 (6)	N2—C28	1.331 (9)
C9—C10	1.570 (6)	N2—C30	1.404 (9)
C10—C11	1.535 (6)	N2—C29	1.436 (9)
C10—H10A	0.9800	C28—H28A	0.9300
C11—C12	1.537 (7)	C29—H29A	0.9600
C11—H11A	0.9700	C29—H29B	0.9600
C11—H11B	0.9700	C29—H29C	0.9600
C12—C16	1.498 (7)	C30—H30A	0.9600
C12—C13	1.542 (7)	C30—H30B	0.9600
C12—H12A	0.9800	C30—H30C	0.9600
C13—C24	1.496 (7)		
			
C21—O2—H2A	109.5	C23—C14—H14A	107.4
C23—O4—H4B	109.5	C13—C14—H14A	107.4
C24—O5—H5A	109.5	C1—C14—H14A	107.4
C15—C1—C2	114.9 (4)	C16—C15—C1	117.3 (4)
C15—C1—C14	107.4 (3)	C16—C15—H15A	121.4
C2—C1—C14	110.6 (4)	C1—C15—H15A	121.4
C15—C1—C10	108.0 (4)	C15—C16—C12	112.5 (4)
C2—C1—C10	111.7 (4)	C15—C16—C17	127.5 (5)
C14—C1—C10	103.5 (4)	C12—C16—C17	120.0 (5)
C3—C2—C1	112.7 (4)	C16—C17—C19	112.2 (6)
C3—C2—H2B	109.0	C16—C17—C18	109.1 (5)
C1—C2—H2B	109.0	C19—C17—C18	110.9 (7)
C3—C2—H2C	109.0	C16—C17—H17A	108.2
C1—C2—H2C	109.0	C19—C17—H17A	108.2
H2B—C2—H2C	107.8	C18—C17—H17A	108.2
C2—C3—C4	110.3 (4)	C17—C18—H18A	109.5
C2—C3—H3A	109.6	C17—C18—H18B	109.5
C4—C3—H3A	109.6	H18A—C18—H18B	109.5
C2—C3—H3B	109.6	C17—C18—H18C	109.5
C4—C3—H3B	109.6	H18A—C18—H18C	109.5
H3A—C3—H3B	108.1	H18B—C18—H18C	109.5
C3—C4—C9	111.0 (4)	C17—C19—H19A	109.5
C3—C4—C5	114.5 (4)	C17—C19—H19B	109.5
C9—C4—C5	117.1 (4)	H19A—C19—H19B	109.5
C3—C4—H4A	104.2	C17—C19—H19C	109.5
C9—C4—H4A	104.2	H19A—C19—H19C	109.5
C5—C4—H4A	104.2	H19B—C19—H19C	109.5
C20—C5—C21	107.0 (5)	C5—C20—H20A	109.5
C20—C5—C6	111.1 (5)	C5—C20—H20B	109.5
C21—C5—C6	110.5 (4)	H20A—C20—H20B	109.5
C20—C5—C4	114.8 (4)	C5—C20—H20C	109.5
C21—C5—C4	105.7 (4)	H20A—C20—H20C	109.5
C6—C5—C4	107.7 (4)	H20B—C20—H20C	109.5
C7—C6—C5	113.2 (4)	O1—C21—O2	122.3 (7)
C7—C6—H6A	108.9	O1—C21—C5	126.7 (7)
C5—C6—H6A	108.9	O2—C21—C5	111.1 (5)
C7—C6—H6B	108.9	C9—C22—H22A	109.5
C5—C6—H6B	108.9	C9—C22—H22B	109.5
H6A—C6—H6B	107.8	H22A—C22—H22B	109.5
C6—C7—C8	111.1 (5)	C9—C22—H22C	109.5
C6—C7—H7A	109.4	H22A—C22—H22C	109.5
C8—C7—H7A	109.4	H22B—C22—H22C	109.5
C6—C7—H7B	109.4	O3—C23—O4	123.9 (5)
C8—C7—H7B	109.4	O3—C23—C14	125.1 (4)
H7A—C7—H7B	108.0	O4—C23—C14	111.0 (5)
C7—C8—C9	113.6 (4)	O6—C24—O5	121.7 (5)
C7—C8—H8A	108.8	O6—C24—C13	124.7 (5)
C9—C8—H8A	108.8	O5—C24—C13	113.6 (5)
C7—C8—H8B	108.8	C25—N1—C26	123.9 (12)
C9—C8—H8B	108.8	C25—N1—C27	124.7 (12)
H8A—C8—H8B	107.7	C26—N1—C27	109.8 (9)
C8—C9—C4	109.3 (4)	O7—C25—N1	145.5 (18)
C8—C9—C22	108.5 (4)	O7—C25—H25A	107.2
C4—C9—C22	112.0 (4)	N1—C25—H25A	107.2
C8—C9—C10	108.2 (4)	N1—C26—H26A	109.5
C4—C9—C10	106.6 (4)	N1—C26—H26B	109.5
C22—C9—C10	112.1 (4)	H26A—C26—H26B	109.5
C11—C10—C9	115.6 (4)	N1—C26—H26C	109.5
C11—C10—C1	108.9 (3)	H26A—C26—H26C	109.5
C9—C10—C1	114.1 (4)	H26B—C26—H26C	109.5
C11—C10—H10A	105.8	N1—C27—H27A	109.5
C9—C10—H10A	105.8	N1—C27—H27B	109.5
C1—C10—H10A	105.8	H27A—C27—H27B	109.5
C10—C11—C12	110.2 (4)	N1—C27—H27C	109.5
C10—C11—H11A	109.6	H27A—C27—H27C	109.5
C12—C11—H11A	109.6	H27B—C27—H27C	109.5
C10—C11—H11B	109.6	C28—N2—C30	120.5 (7)
C12—C11—H11B	109.6	C28—N2—C29	121.0 (7)
H11A—C11—H11B	108.1	C30—N2—C29	118.0 (7)
C16—C12—C11	108.8 (4)	O8—C28—N2	124.9 (6)
C16—C12—C13	107.8 (4)	O8—C28—H28A	117.6
C11—C12—C13	109.0 (4)	N2—C28—H28A	117.6
C16—C12—H12A	110.4	N2—C29—H29A	109.5
C11—C12—H12A	110.4	N2—C29—H29B	109.5
C13—C12—H12A	110.4	H29A—C29—H29B	109.5
C24—C13—C12	112.9 (4)	N2—C29—H29C	109.5
C24—C13—C14	111.8 (4)	H29A—C29—H29C	109.5
C12—C13—C14	108.3 (4)	H29B—C29—H29C	109.5
C24—C13—H13A	107.9	N2—C30—H30A	109.5
C12—C13—H13A	107.9	N2—C30—H30B	109.5
C14—C13—H13A	107.9	H30A—C30—H30B	109.5
C23—C14—C13	111.9 (4)	N2—C30—H30C	109.5
C23—C14—C1	111.8 (4)	H30A—C30—H30C	109.5
C13—C14—C1	110.6 (4)	H30B—C30—H30C	109.5
			
C15—C1—C2—C3	74.5 (5)	C16—C12—C13—C14	59.5 (5)
C14—C1—C2—C3	−163.6 (4)	C11—C12—C13—C14	−58.5 (5)
C10—C1—C2—C3	−49.0 (5)	C24—C13—C14—C23	104.6 (5)
C1—C2—C3—C4	57.9 (5)	C12—C13—C14—C23	−130.4 (4)
C2—C3—C4—C9	−65.1 (5)	C24—C13—C14—C1	−130.0 (4)
C2—C3—C4—C5	159.6 (4)	C12—C13—C14—C1	−5.0 (5)
C3—C4—C5—C20	58.3 (6)	C15—C1—C14—C23	76.2 (5)
C9—C4—C5—C20	−74.1 (6)	C2—C1—C14—C23	−49.9 (5)
C3—C4—C5—C21	−59.3 (6)	C10—C1—C14—C23	−169.6 (4)
C9—C4—C5—C21	168.3 (4)	C15—C1—C14—C13	−49.2 (5)
C3—C4—C5—C6	−177.4 (4)	C2—C1—C14—C13	−175.3 (4)
C9—C4—C5—C6	50.2 (6)	C10—C1—C14—C13	65.0 (4)
C20—C5—C6—C7	72.7 (6)	C2—C1—C15—C16	178.8 (4)
C21—C5—C6—C7	−168.8 (5)	C14—C1—C15—C16	55.3 (5)
C4—C5—C6—C7	−53.8 (6)	C10—C1—C15—C16	−55.8 (5)
C5—C6—C7—C8	58.7 (6)	C1—C15—C16—C12	0.1 (6)
C6—C7—C8—C9	−56.7 (6)	C1—C15—C16—C17	−178.0 (5)
C7—C8—C9—C4	50.2 (6)	C11—C12—C16—C15	58.1 (5)
C7—C8—C9—C22	−72.2 (5)	C13—C12—C16—C15	−59.9 (6)
C7—C8—C9—C10	165.9 (4)	C11—C12—C16—C17	−123.6 (5)
C3—C4—C9—C8	177.1 (4)	C13—C12—C16—C17	118.4 (5)
C5—C4—C9—C8	−48.9 (5)	C15—C16—C17—C19	−12.9 (9)
C3—C4—C9—C22	−62.6 (5)	C12—C16—C17—C19	169.1 (6)
C5—C4—C9—C22	71.4 (5)	C15—C16—C17—C18	110.4 (7)
C3—C4—C9—C10	60.3 (5)	C12—C16—C17—C18	−67.6 (8)
C5—C4—C9—C10	−165.6 (4)	C20—C5—C21—O1	−4.4 (8)
C8—C9—C10—C11	63.0 (5)	C6—C5—C21—O1	−125.5 (7)
C4—C9—C10—C11	−179.6 (4)	C4—C5—C21—O1	118.3 (7)
C22—C9—C10—C11	−56.7 (5)	C20—C5—C21—O2	176.4 (5)
C8—C9—C10—C1	−169.6 (4)	C6—C5—C21—O2	55.3 (6)
C4—C9—C10—C1	−52.1 (5)	C4—C5—C21—O2	−60.9 (6)
C22—C9—C10—C1	70.8 (5)	C13—C14—C23—O3	43.9 (6)
C15—C1—C10—C11	50.7 (5)	C1—C14—C23—O3	−80.8 (6)
C2—C1—C10—C11	178.0 (4)	C13—C14—C23—O4	−137.1 (4)
C14—C1—C10—C11	−63.1 (5)	C1—C14—C23—O4	98.2 (5)
C15—C1—C10—C9	−80.1 (5)	C12—C13—C24—O6	−110.1 (6)
C2—C1—C10—C9	47.2 (5)	C14—C13—C24—O6	12.2 (7)
C14—C1—C10—C9	166.1 (4)	C12—C13—C24—O5	68.2 (6)
C9—C10—C11—C12	132.6 (4)	C14—C13—C24—O5	−169.5 (4)
C1—C10—C11—C12	2.6 (5)	C26—N1—C25—O7	−84 (3)
C10—C11—C12—C16	−57.0 (5)	C27—N1—C25—O7	112 (3)
C10—C11—C12—C13	60.4 (5)	C30—N2—C28—O8	−175.9 (7)
C16—C12—C13—C24	−176.1 (4)	C29—N2—C28—O8	−4.6 (12)
C11—C12—C13—C24	65.9 (5)		

### Hydrogen-bond geometry (Å, °)

**Table:**

*D*—H···*A*	*D*—H	H···*A*	*D*···*A*	*D*—H···*A*
O2—H2A···O7^i^	0.82	1.85	2.653 (8)	168
O4—H4B···O8^ii^	0.82	1.74	2.549 (6)	168
O5—H5A···O3^iii^	0.82	1.96	2.781 (5)	176

Symmetry codes: (i) *x*+1/2, −*y*+1/2, −*z*; (ii) *x*+1, *y*, *z*; (iii) *x*−1, *y*, *z*.

## References

[bb1] Aldrich, P. H. (1971). US Patent No. 3 562 243.

[bb2] Enraf–Nonius (1994). *CAD-4 EXPRESS* Enraf–Nonius, Delft, The Netherlands.

[bb3] Farrugia, L. J. (1997). *J. Appl. Cryst.* **30**, 565.

[bb5] Halbrook, N. J. & Lawrence, R. V. (1958). *J. Am. Chem. Soc.* **80**, 368–370.

[bb6] Halbrook, N. J. & Lawrence, R. V. (1959). US Patent No. 2 889 362.

[bb7] Harms, K. & Wocadlo, S. (1995). *XCAD4* University of Marburg, Germany.

[bb10] North, A. C. T., Phillips, D. C. & Mathews, F. S. (1968). *Acta Cryst.* A**24**, 351–359.

[bb8] Sheldrick, G. M. (2008). *Acta Cryst.* A**64**, 112–122. 10.1107/S0108767307043930 18156677

[bb9] Song, Z. Q., Xu, X., Shang, S. B., Wang, H. X. & Rao, X. P (2009). Chinese Patent CN 101591239.

